# Non-HLA genes PTPN22, CDK6 and PADI4 are associated with specific autoantibodies in HLA-defined subgroups of rheumatoid arthritis

**DOI:** 10.1186/s13075-014-0414-3

**Published:** 2014-08-20

**Authors:** Omri Snir, David Gomez-Cabrero, Ariana Montes, Eva Perez-Pampin, Juan J Gómez-Reino, Maria Seddighzadeh, Katharina U Klich, Lena Israelsson, Bo Ding, Anca I Catrina, Rikard Holmdahl, Lars Alfredsson, Lars Klareskog, Jesper Tegnér, Antonio Gonzalez, Vivianne Malmström, Leonid Padyukov

**Affiliations:** Rheumatology Unit, Department of Medicine at Karolinska University Hospital, Karolinska Institute, Solna, CMM L8:O4 Karolinska Hospital, Stockholm, SE-171 76 Sweden; Computational Medicine Unit, Department of Medicine at Karolinska University Hospital, Karolinska Institute, Solna, CMM L8:O5 Karolinska Hospital, Stockholm, SE-171 76 Sweden; Laboratorio de Investigacion 10, Instituto de Investigacion Sanitaria–Hospital Clinico Universitario de Santiago, Travesia de Choupana sn, Santiago de Compostela, ES-157 06 Spain; Institute of Environmental Medicine, Karolinska Institutet, Nobels väg 13, Stockholm, SE-171 77 Sweden; Division of Medical Inflammation Research, Department of Medical Biochemistry and Biophysics, Karolinska Institutet, Scheeles väg 2, B2, Stockholm, SE-171 77 Sweden

## Abstract

**Introduction:**

Genetic susceptibility to complex diseases has been intensively studied during the last decade, yet only signals with small effect have been found leaving open the possibility that subgroups within complex traits show stronger association signals. In rheumatoid arthritis (RA), autoantibody production serves as a helpful discriminator in genetic studies and today anti-citrullinated cyclic peptide (anti-CCP) antibody positivity is employed for diagnosis of disease. The *HLA-DRB1* locus is known as the most important genetic contributor for the risk of RA, but is not sufficient to drive autoimmunity and additional genetic and environmental factors are involved. Hence, we addressed the association of previously discovered RA loci with disease-specific autoantibody responses in RA patients stratified by HLA-DRB1*04.

**Methods:**

We investigated 2178 patients from three RA cohorts from Sweden and Spain for 41 genetic variants and four autoantibodies, including the generic anti-CCP as well as specific responses towards citrullinated peptides from vimentin, alpha-enolase and type II collagen.

**Results:**

Our data demonstrated different genetic associations of autoantibody-positive disease subgroups in relation to the presence of DRB1*04. Two specific subgroups of autoantibody-positive RA were identified. The SNP in *PTPN22* was associated with presence of anti-citrullinated enolase peptide antibodies in carriers of HLA-DRB1*04 (Cochran-Mantel-Haenszel test *P* = 0.0001, *P*_corrected_ <0.05), whereas SNPs in *CDK6* and *PADI4* were associated with anti-CCP status in DRB1*04 negative patients (Cochran-Mantel-Haenszel test *P* = 0.0004, *P*_corrected_ <0.05 for both markers). Additionally we see allelic correlation with autoantibody titers for *PTPN22* SNP rs2476601 and anti-citrullinated enolase peptide antibodies in carriers of HLA-DRB1*04 (Mann Whitney test *P* = 0.02) and between *CDK6* SNP rs42041 and anti-CCP in non-carriers of HLA-DRB1*04 (Mann Whitney test *P* = 0.02).

**Conclusion:**

These data point to alternative pathways for disease development in clinically similar RA subgroups and suggest an approach for study of genetic complexity of disease with strong contribution of HLA.

**Electronic supplementary material:**

The online version of this article (doi:10.1186/s13075-014-0414-3) contains supplementary material, which is available to authorized users.

## Introduction

The study of complex diseases has revealed complicated patterns of inheritance [1] in which involvement of multiple variants and environmental conditions, as well as gene-gene and gene-environmental interactions, have made the discovery of genetic causes for complex diseases a challenging task. One main methodological caveat is the definition of phenotypes. As clinical features of complex diseases are heterogeneous, one cannot expect simple genetic patterns to explain such complex heterogeneity. By stratifying for more homogeneous disease subgroups/phenotypes one may try to achieve stronger association and better understanding of disease mechanisms [2,3].

Rheumatoid arthritis (RA) is a common complex disease with incidence around 0.5 to 1.0% in different populations [4,5] and a steadily growing prevalence in aging Western and Eastern societies. Due to implementation of several targeted treatments in combination with immunosuppressive therapy the overall quality of life for RA patients has dramatically improved and many patients may stay in remission for long periods of time [6]. However, current treatments mostly decelerate rather than cure disease, while disease-driving mechanisms remain poorly understood [6,7]. Despite the discovery of multiple genetic associations with RA, variants within the *HLA-DRB1* locus, (that is, HLA-DRB1*04 alleles), remain the most significant contributors to the risk of autoantibody-positive RA [8–14].

Antibody responses against citrullinated epitopes of vimentin, fibrinogen, type-II collagen, alpha-enolase represent specific features of RA [11,12,15,16]. Patients may display antibodies to one or several of these modified self-proteins. Most of these autoantibody specificities are confined within, and could thus be recognized on testing as anti-citrullinated cyclic peptide (CCP) antibodies [11,12], which is a generic test recognizing most anti-citrulinated peptide antibody (ACPAs). A strong association between the shared epitope (SE) *HLA-DRB1* alleles, specifically for SE HLA-DRB1*04 alleles, and development of both anti-CCP and anti-citrullinated alpha-enolase peptide-1 (CEP-1) antibodies has been reported [17]. Subsets of RA defined by other combinations of antibodies to citrullinated autoantigens have been shown to display very different degrees of association with the common *HLA-DRB1* risk alleles that constitute the group of shared epitope alleles [18,19]. These different profiles of ACPAs may reflect distinct biological and immunological courses that are determined by genetic niches of susceptibility. Although it is clinically challenging to differentiate these subgroups due to rather similar symptoms, it is essential to dissect these in view of diagnosis and treatment, and ultimately for the understanding of disease mechanisms and possible prevention.

To find additional links between genetics and serology of RA, which will allow for studies of the relationship between genotypes and phenotypes, we employed a large population-based study from Sweden, the Epidemiological Investigation of Rheumatoid Arthritis (EIRA) with incident cases of RA and two smaller cohorts of cases with well-established/chronic RA. We also considered known relative stability of anti-CCP levels through the RA development [20,21]. Our hypothesis is that a polymorphism outside the *HLA-DRB1* locus may contribute and shape the development of certain serological subtypes of RA, which are otherwise clinically indistinguishable. We found that contribution of different non-HLA single nucleotide polymorphisms (SNPs) associated with RA in the development of distinct ACPAs does not overlap, and may define distinct subgroups of disease with an SE-positive or an SE-negative background.

## Methods

### Patients and healthy subjects

DNA and serum samples were collected from three independent cohorts from Sweden and Spain (Additional file 1: Table S13); all experiments were performed in accordance with the Declaration of Helsinki and were approved by Stockholm Ethical Review board or *Comite Etico de Investigacion Clinica de Galicia* and all subjects gave informed consent. Serum and DNA samples were stored at −80°C until use. This was a case-case study to test the hypothesis of the contribution of different genetic factors in the development of serologically determined subgroups of RA.

The cohorts included were: 1) For initial study we analyzed a cohort of 1,362 patients with incident RA (cohort 1) from a population-based case–control study (EIRA) [9,22]. The details of the EIRA study have been described previously [22]. Briefly, a case was defined as a person in the study base who received a new diagnosis of RA from a rheumatologist (within 1 year after the onset of symptoms in 85% of the cases) and fulfilled the American College of Rheumatology 1987 criteria for the classification of RA [23]. Cases were recruited from all public and a majority of private rheumatology units in the study area; 2) cohort 2 comprised 379 patients with established RA, who all fulfilled the American College of Rheumatology criteria [23] and were attending the Rheumatology Clinic at the Karolinska University Hospital, Stockholm, Sweden; 3) cohort 3 comprised 437 patients with established RA classified according to the 1987 American College of Rheumatology criteria and of Spanish ancestry; DNA and serum samples from these patients were obtained from a single hospital. Their clinical characteristics have been already described in full [24].

### Serologic measurement

#### Detection of IgG anti-CCP antibodies

ELISA Anti-CCP2 test (Immunoscan RA, Mark 2, Euro-Diagnostica, Malmö, Sweden) was used to determine the levels of anti-CCP IgG antibodies. Quantification of the results and use of a cut-off value of 25 U/ml were according to the manufacturer’s instructions.

#### Detection of IgG antibodies against citrullinated alpha-enolase peptide-1 (CEP-1, citrullinated type-II collagen (citC1^III^) and citrullinated vimentin (cit-vim)

ELISA for detection of IgG antibodies against CEP-1, citC1^III^ and cit-Vim was preformed as previously described [12,19,24].

### Genotyping

#### HLA-DR genotyping

DNA was extracted from ethylenediaminetetraacetic acid (EDTA)-treated blood by the salting-out method [25]. Genotyping for *HLA-DRB1* allotypes was conducted using the sequence-specific primer-PCR method (DR low-resolution kit; Olerup SSP, Saltsjöbaden, Sweden) as previously described [26]. DRB1*04- and DRB1*01-positive patients were further subtyped by Olerup SSP DRB1*04 (Olerup SSP) subtyping kits, respectively [27,28]. In replication cohort 3, *HLA-DBR1* alleles were genotyped by a sequencing-based typing method using the AlleleSEQR HLA-DRB1 Typing kit (Abbott Diagnostics, Abbott Park, Germany), which includes bidirectional sequencing of the second exon of DRB1. Ambiguous samples were additionally sequenced with group-specific primers (AlleleSEQR HLA-DRB1 GSSP, Abbott Diagnostics, Abbott Park, Germany).

#### Genotyping for RA-predisposing allelic variants

The 41 SNPs in the first phase of this study were selected from previous studies of genetic risk factors for RA; references for each SNP are presented in Additional file 1: Table S14. These 41 SNPs were studied in the two first cohorts of patients. Genotypes corresponding to the EIRA cohort were retrieved from a genome-wide association study (GWAS) (26 SNPs) [29], from dense mapping of the HLA region (4 SNPs) [30] or from additional genotyping (11 SNPs) that was performed by TaqMan allelic discrimination assay (Applied Biosystems, Foster City, CA, USA). Genotyping of cohort-2 was performed using a 64-OpenArray platform (Applied Biosystems) with the chip-based TaqMan genotyping technology. Genotyping was performed according to manufacturer’s instructions, and genotype calls were made using AutoCaller (Applied Biosystems). All SNPs that were analyzed in this study are listed in Additional file 1: Table S14.

With the results of the two first cohorts we selected 12 SNPs for further analysis. The criteria of selection were either a *P*-value <0.01 for association of the SNP with a specific subgroup of autoantibody-positive patients in any of the two cohorts, or to show the same direction of association and a *P*-value <0.2 in the two cohorts. Genotyping for the 12 selected SNPs in cohort 3 (Spanish patients) was performed by PCR amplification followed by single-base extension with the SNaPshot Multiplex Kit (Applied Biosystems). Details of the protocol are available by the authors upon request. SNP rs2476601 was genotyped with a Taqman SNP genotyping assay (Applied Biosystems, Carlsbad, CA, USA).

### Statistical analyses

We performed analyses either for the whole patient cohort, or for separate HLA-DRB1*04-positive patients and HLA-DRB1*04-negative patients and further calculated the odds ratios (OR) and 95% CI for the association with anti-CCP and for three ACPA specificities. A dominant genetic model or carrier model that assess the effect of the presence of the risk allele, irrespective of the presence of one or two copies, was used for all analyses to avoid too few counts of some patient subgroups. The OR were calculated by median-unbiased estimation and exact CI using the mid-*P* method [31] with patients without the specific type of antibody as the reference group.

The three cohorts were jointly analyzed (citC1^III^ data were only available for two cohorts) using the Cochran-Mantel-Haenszel (CMH) test. An adjusted *P*-value of 0.05 after Bonferroni correction for multiple testing was considered statistically significant in the meta-analysis. Homogeneity between cohorts was tested by Tarone's test. As we detected no significant heterogeneity between cohorts a CMH fixed-effect model was fitted using the restricted maximum-likelihood estimator. The *metafor* R package was used for these analyses [32]. Antibody levels and disease activity score in 28 joints (DAS28) were compared by the Mann–Whitney test.

## Results

**Figure 1:**
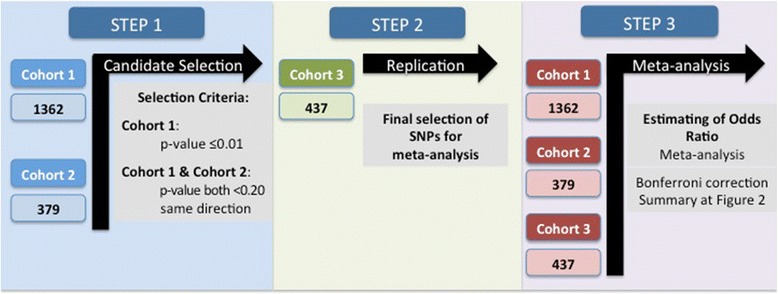
**Visual description of the analysis.** SNP, single nucleotide polymorphism.

We determined the associations between genotypes and phenotypes by performing a three-step analysis that is represented in the analytical workflow chart in Figure 1. At the first step, by analysis of cohort 1 and replication in cohort 2, we selected 12 SNPs for further replication. In a second step we validated the results in an independent cohort (cohort 3) and in the third step we estimated OR for RA with certain serologic profiles, based on data from all three cohorts, by meta-analysis. Our main goal was to detect possible associations of non-DRB1 SNPs with autoantibody-defined subgroups in relation to presence/absence of HLA-DRB1*04 alleles. However, we also performed analysis for the whole group of patients without this stratification.

### Association study in cohort 1 and cohort 2

Cohort 1 comprised 1,362 RA patients from the Swedish EIRA study: 70% of the patients were positive for anti-CCP antibodies, whereas defined ACPA fine specificities were less frequent; 50% of the patients were positive for anti-CEP-1 and 42% were positive for anti-cit-Vim and for anti-citC1^III^ antibodies (Additional file 1: Table S13). We performed a case-case association study to test the hypothesis of different genetic backgrounds between RA subgroups identified by autoantibody specificity. Multiple SNPs were associated in the whole cohort or after stratification by the major SE allele, HLA-DRB1*04, but only few passed conservative corrections for multiple testing (*P* ≤0.0004, Additional file 1: Tables S1-S4), namely: SNP rs6457617 (*HLA-DQB1* gene) for several specificities, for the whole cohort and also after stratification for HLA-DRB1*04, and rs2476601 (*PTPN22* gene) for the HLA-DRB1*04-positive RA group with anti-CEP-1 autoantibody.

With the goal to gain better statistical power and to validate the associations identified, we performed analysis in cohort 2, an independent cohort of 379 Swedish RA patients with established disease (Additional file 1: Table S13). Most patients in this cohort were positive for anti-CCP (73%) and for anti-citVim (58%). The other two ACPAs were less frequent (46% for anti-CEP-1 and 38% for anti-citC1^III^). Again, although multiple SNPs demonstrated a moderate trend towards association, very few passed correction for multiple comparisons (Additional file 1: Tables S5-S8). Therefore, we choose to select the best candidate SNPs from association studies in cohorts 1 and 2 for further validation. Two criteria were used for selection, either a *P*-value <0.01 in any of the two cohorts, or a change in the same direction with *P* <0.2 in the two cohorts for the same comparison. This procedure generated a list of 12 SNPs that were used at the next step. Extracted data for association with these SNPs from analyses of cohorts 1 and 2 are presented in Table 1.

**Table 1:** **Selected associations found in cohorts 1 and 2**

**Gene**	**rs number**	**Chromosome**	**Position**	**Cohort 1** ***P*** **-value**	**Cohort 1 odds ratio (95% CI)**	**Cohort 2** ***P*** **-value**	**Cohort 2 odds ratio (95% CI)**
**Anti-CCP**							
**Anti-CCP - all patients**							
*PTPN22*	rs2476601	1	114179091	0.119	1.235 (0.948, 1.617)	0.104	1.519 (0.920, 2.569)
*MICA*	rs2523451	6	31477130	0.062	1.382 (0.984, 1.938)	0.035	1.707 (1.039, 2.795)
*HLA-DQB1*	rs6457617	6	32771829	2.22 × 10^−16^	4.232 (2.998, 6.008)	0.131	2.028 (0.803, 4.911)
*CDK6*	rs42041	7	92084680	0.017	1.368 (1.056, 1.777)	0.162	1.404 (0.874, 2.285)
**Anti-CCP-–** HLA-DRB1*04 **patients**							
*PTPN22*	rs2476601	1	114179091	0.018	1.67 (1.090, 2.628)	0.19	1.751 (0.765, 4.415)
**Anti-CCP - Non-** HLA-DRB1*04 **patients**							
*PADI4*	rs2240340	1	17535226	0.012	1.718 (1.128, 2.625)	0.15	1.627 (0.838, 3.176)
*CDK6*	rs42041	7	92084680	0.001	1.831 (1.265, 2.660)	0.055	1.909 (0.987, 3.772)
**Citrullinated-vimentin**							
**Citrullinated-vimentin - all patients**							
***PTPN22***	**rs2476601**	**1**	**114179091**	**0.048**	**1.272 (1.002, 1.614)**	**0.021**	**1.691 (1.083, 2.667)**
***MICA***	**rs2523451**	**6**	**31477130**	**0.005**	**1.595 (1.151, 2.221)**	**0.009**	**1.818 (1.162, 2.851)**
***HLA-DQB1***	**rs6457617**	**6**	**32771829**	**2.36** × **10** ^**−10**^	**3.417 (2.285, 5.266)**	**0.001**	**4.948 (1.890, 15.652)**
*HLA-DPB1*	rs2064476	6	33181300	0.19	1.636 (0.787, 3.594)	0.028	3.228 (1.132, 10.682)
*TRAF1*	rs3761847	9	122730060	0.088	1.254 (0.967, 1.631)	0.171	1.406 (0.863, 2.289)
**Citrullinated-vimentin -** HLA-DRB1*04 **patients**							
***PTPN22***	**rs2476601**	**1**	**114179091**	**0.022**	**1.437 (1.054, 1.967)**	**0.04**	**1.912 (1.029, 3.672)**
*MICA*	rs2523451	6	31477130	0.085	1.495 (0.945, 2.362)	0.133	1.639 (0.858, 3.106)
**Citrullinated-vimentin - non-** HLA-DRB1*04 **patients**							
*HLA-DQB1*	rs6457617	6	32771829	0.161	1.387 (0.880, 2.233)	0.042	2.814 (1.034, 9.151)
**CEP-1**							
**CEP-1- all patients**							
*PTPN22*	rs2476601	1	114179091	0.016	1.339 (1.055, 1.702)	0.078	1.475 (0.957, 2.279)
*HLA-DQB1*	rs6457617	6	32771829	5.11 × 10^−11^	3.213 (2.242, 4.676)	0.166	1.896 (0.772, 5.144)
*PRKCQ*	rs4750316	10	6433266	0.133	1.204 (0.945, 1.534)	0.004	1.86 (1.220, 2.849)
*HTR2A*	rs1328674	13	46339708	0.187	1.33 (0.871, 2.051)	0.115	1.924 (0.854, 4.546)
**CEP-1 -** HLA-DRB1*04 **patients**							
*CIITA*	rs6416647	16	10873098	0.011	1.525 (1.099, 2.121)	0.194	1.431 (0.833, 2.468)
**CEP-1 - non-** HLA-DRB1*04 **patients**							
*HLA-DQB1*	rs2064476	6	33181300	0.002	1.894 (1.268, 2.865)	0.155	3.8 (0.656, 97.488)
*PRKCQ*	rs4750316	10	6433266	0.106	1.379 (0.934, 2.035)	0.003	2.919 (1.446, 5.965)
**CitC1** ^**III**^							
**CitC1** ^**III**^ **- all patients**							
*MMEL1*	rs3890745	1	2543484	0.158	1.319 (0.899, 1.959)	0.122	1.883 (0.850, 4.634)
*CDK6*	rs42041	7	92084680	0.166	1.181 (0.933, 1.495)	0.044	1.549 (1.012, 2.372)
**CitC1 -** HLA-DRB1*04 **patients**							
*TNFAIP3*	rs6920220	6	138048197	0.067	1.796 (0.960, 3.504)	0.052	3.254 (0.991, 15.257)
**CitC1** ^**III**^ **- non-** HLA-DRB1*04 **patients**							
*CDK6*	rs42041	7	92084680	0.038	1.502 (1.023, 2.210)	0.051	1.99 (0.997, 3.988)

P-values in bold represent the most significant associations with values <0.05 for both cohorts.

### Association study in cohort 3

The 12 SNPs were genotyped in an independent collection of patients of Spanish ancestry, who had established RA (n = 437). They showed a similar fraction of anti-CCP positivity, but a lower frequency of the ACPAs (Additional file 1: Table S13). The analysis revealed moderate levels of association for SNPs from *PTPN22*, *PADI4*, *CDK6* and *MICA* in some disease subgroups (Additional file 1: Tables S9-S11). Importantly, the direction of associations followed the same pattern as in cohorts 1 and 2.

### Meta-analysis of the three cohorts

**Figure 2:**
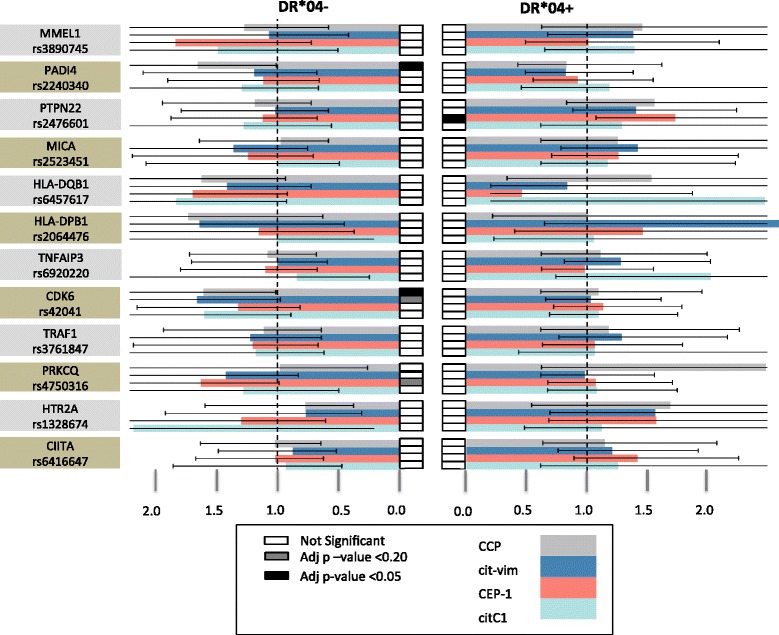
**Overview of the most significant associations in the study.** The bars represent the odds ratio on the horizontal scale with the 99.999% CI for comparison between group of persons with and without autoantibody (distinguished by color of the bar) and carriers and non-carriers of the major allele with separation to HLA-DRB1*04-positive and HLA-DRB1*04-negative. Results are presented for the final selection of SNPs and meta-analysis of three cohorts (two cohorts for anti-CitC1). Indicated *P*-values are the values after Bonferroni correction. Full details are presented in Additional file 1: Table S12.

Following examination for heterogeneity, a fixed-effect meta-analysis was conducted for the association of these SNPs with anti-citrulline immunity in RA using data available from the three cohorts; Table 2 and Figure 2 provide summarized results, which were Bonferroni-corrected for the number of tests done. Additional file 1: Table S12 provides the full disclosure of the analysis.

**Table 2:** **Association of genetic markers with serological subgroups of rheumatoid arthritis: meta-analysis summary**

**Anti-CPP**					
		HLA-DRB1*04 **-positive group**		HLA-DRB1*04 **-negative group**	
**Reference sequence for single nucleotide polymorphism (SNP)**	**Gene**	**Uncorrected** ***P*** **-value**	**Corrected** ***P*** **-value**	**Uncorrected** ***P*** **-value**	**Corrected** ***P*** **-value**
rs3890745	*MMEL1*	0.1222	1.000	0.2888	1.000
rs2240340	*PADI4*	0.3381	1.000	**0.0004**	**0.038**
rs2476601	*PTPN22*	0.0142	1.000	0.2356	1.000
rs2523451	*MICA*	0.2676	1.000	0.8643	1.000
rs6457617	*HLA-DQ*	0.3272	1.000	0.0024	0.228
rs2064476	*HLA.DPB2*	0.9817	1.000	0.0604	1.000
rs6920220	*TNFAIP3*	0.5221	1.000	0.5579	1.000
rs42041	*CDK6*	0.5768	1.000	**0.0004**	**0.034**
rs3761847	*TRAF1*	0.3759	1.000	0.5073	1.000
rs4750316	*PRKCQ*	0.0225	1.000	0.9611	1.000
rs1328674	*HTR2A*	0.1093	1.000	0.2167	1.000
rs6416647	*CIITA*	0.4205	1.000	0.8600	1.000
**anti-cit-Vimentin**					
		HLA-DRB1*04 **-positive group**		HLA-DRB1*04 **-negative group**	
**Reference sequence for SNP**	**Gene**	**Uncorrected** ***P*** **-value**	**Corrected** ***P*** **-value**	**Uncorrected** ***P*** **-value**	**Corrected** ***P*** **-value**
rs3890745	*MMEL1*	0.1174	1.000	0.8047	1.000
rs2240340	*PADI4*	0.1918	1.000	0.2833	1.000
rs2476601	*PTPN22*	0.0111	1.000	0.9087	1.000
rs2523451	*MICA*	0.0394	1.000	0.0713	1.000
rs6457617	*HLA-DQ*	0.6668	1.000	0.0727	1.000
rs2064476	*HLA.DPB2*	0.0172	1.000	0.1853	1.000
rs6920220	*TNFAIP3*	0.0601	1.000	0.9923	1.000
rs42041	*CDK6*	0.8207	1.000	**0.0009**	**0.088**
rs3761847	*TRAF1*	0.0877	1.000	0.2822	1.000
rs4750316	*PRKCQ*	0.8846	1.000	0.0231	1.000
rs1328674	*HTR2A*	0.0557	1.000	0.3165	1.000
rs6416647	*CIITA*	0.1542	1.000	0.3807	1.000
**anti-CEP-1**					
		HLA-DRB1*04 **-positive group**		HLA-DRB1*04 **-negative group**	
**Reference sequence for SNP**	**Gene**	**Uncorrected** ***P*** **-value**	**Corrected** ***P*** **-value**	**Uncorrected** ***P*** **-value**	**Corrected** ***P*** **-value**
rs3890745	*MMEL1*	0.9475	1.000	0.0240	1.000
rs2240340	*PADI4*	0.6073	1.000	0.4651	1.000
rs2476601	*PTPN22*	**0.0001**	**0.006**	0.4364	1.000
rs2523451	*MICA*	0.1623	1.000	0.1834	1.000
rs6457617	*HLA-DQ*	0.0558	1.000	0.0028	0.267
rs2064476	*HLA.DPB2*	0.3083	1.000	0.6594	1.000
rs6920220	*TNFAIP3*	0.8999	1.000	0.5037	1.000
rs42041	*CDK6*	0.3235	1.000	0.0459	1.000
rs3761847	*TRAF1*	0.6790	1.000	0.2747	1.000
rs4750316	*PRKCQ*	0.5904	1.000	**0.0007**	**0.070**
rs1328674	*HTR2A*	0.0582	1.000	0.2357	1.000
rs6416647	*CIITA*	0.0089	0.852	0.8989	1.000
**anti- CitC1** ^**III**^					
		HLA-DRB1*04 **-positive group**		HLA-DRB1*04 **-negative group**	
**Reference sequence for SNP**	**Gene**	**Uncorrected** ***P*** **-value**	**Corrected** ***P*** **-value**	**Uncorrected** ***P*** **-value**	**Corrected** ***P*** **-value**
rs3890745	*MMEL1*	0.1310	1.000	0.2013	1.000
rs2240340	*PADI4*	0.5328	1.000	0.1821	1.000
rs2476601	*PTPN22*	0.2301	1.000	0.3068	1.000
rs2523451	*MICA*	0.3840	1.000	0.9666	1.000
rs6457617	*HLA-DQ*	0.4136	1.000	0.0021	0.201
rs2064476	*HLA.DPB2*	0.9029	1.000	0.9872	1.000
rs6920220	*TNFAIP3*	0.0148	1.000	0.6307	1.000
rs42041	*CDK6*	0.4844	1.000	0.0054	0.522
rs3761847	*TRAF1*	0.8086	1.000	0.3773	1.000
rs4750316	*PRKCQ*	0.5519	1.000	0.3662	1.000
rs1328674	*HTR2A*	0.6374	1.000	0.3458	1.000
rs6416647	*CIITA*	0.2613	1.000	0.7296	1.000

Odds ratios are presented in Figure 2; full details are presented in Additional file 1: Table S12. *P*-values in bold represent the most significant associations with corrected values <0.10.

The results following stratification into carriers of HLA-DRB1*04 and non-carriers of HLA-DRB1*04 showed associations of the SNP in *PTPN22* with antibodies to CEP-1 in the HLA-DRB1*04-positive group (adjusted *P*-value 0.007), and of two SNPs, in *PADI4* and in *CDK6*, with anti-CCP response in the HLA-DRB1*04-negative group (adjusted p-value 0.038 and 0.034, respectively). In addition, two SNPs showed a trend to association with *P*-value <0.1 in the non-carriers of HLA-DRB1*04: *CDK6* and *PRKCQ* with anti-citVim and anti-CEP-1 respectively (Table 2, Additional file 1: Table S12, and Figure 2). These data additionally endorse the association of RA subgroups with the *CDK6* polymorphism and suggest *PRKCQ* as a relevant candidate for follow-up studies.

In the analyses without stratification according to HLA-DRB1*04, the SNP assigned to *MICA* was associated with response to cit-Vim (adjusted *P*-value 0.008), the *PTPN22* SNP was associated with antibodies to CEP-1 (adjusted *P*-value 0.012), and the *HLA-DQB1* SNP was associated with the anti-citC1^III^ antibodies (adjusted *P* = 9 × 10^−5^) (Additional file 1: Table S12). These findings suggest a relative independence of the association with the *MICA* SNP from HLA-DRB1*04 as the OR are very uniform in the two groups of patients stratified by this allele (OR = 1.4 in the carriers and in non-carriers). This independence was not present in the association with the *PTPN22* SNP that showed a stronger significant association in the carriers of DRB1*04 than in the non-carriers (OR = 1.7 and 1.1, respectively).

### Analysis of serological and clinical data

As our data showed that SNPs in *PADI4*, *PTPN22* and *CDK6* together with HLA-DRB1*04 carrier status were associated with the presence of some autoantibodies, we tested if the genotypes were correlated with the titers of these antibodies. We saw a coherent significant difference for anti-CCP antibody levels between homozygous CC patients and those carrying the G allele of rs42041 from *CDK6* in cohort 1 and 2 for patients without DRB1*04 alleles (*P* = 0.0193 and 0.0290 for cohorts 1 and 2, Additional file 2: Figure S1a). Similarly, patients having the AA/AG variant of rs2476601 from *PTPN22* had higher levels of antibodies towards cit-Vim in cohort 1 and 2 but only in patients with DRB1*04 alleles (*P* = 0.0238, Additional file 2: Figure S1c).

To compare genetic subgroups defined by certain autoantibody specificity, HLA-DRB1*04 and non-HLA alleles (*PTPN22*, *PADI4* and *CDK6*), we analyzed data on disease activity at baseline in cohort 1 measured by the DAS28, but no differences were found (data not shown).

## Discussion

In this study we have demonstrated that specificity of autoantibody responses in seropositive RA is regulated not only by HLA-DRB1*04, but also by at least three additional non-HLA genetic variants: *PADI4*, *PTPN22* and *CDK6*. Our data suggest that *PTPN22* involvement is restricted to HLA-DRB1*04 carriers, while associations of *PADI4* and *CDK6* are restricted to the non-carriers, and they also differ in autoantibody specificity. While concordance of HLA-DRB1*04 and *PTPN22* SNP in anti-CEP-1 positive RA was demonstrated previously in a single cohort [17,19], the involvement of genetic variants of *PADI4* and *CDK6* in risk of development of RA subgroups only in the absence of HLA-DRB1*04 is a new finding. We also have observed a difference in the autoantibody titers corresponding with the previous associations, (Additional file 2: Figure S1) while no difference was found in relation to clinical activity of disease at baseline measured by the DAS28.

Genetic and environmental factors are involved in the etiology of complex diseases. In RA, such susceptibility factors have been primarily found for ACPA-positive disease [7,9,33]. Among RA-predisposing genes, the involvement of the *HLA-DRB1* polymorphism has been extensively studied, as it was the first identified and it was also the strongest to date [27,34]. Association of the shared epitope alleles of *HLA-DRB1* with ACPA (anti-CCP)-positive RA indicates probable mechanisms for disease development. In this work, we further examined the significance of other, mainly non-HLA variants, for the development of different autoantibodies in a case-case study. We investigated the effects of these genetic variants alone or in combination with HLA-DRB1*04, the most frequent SE alleles in European Caucasians, in three independent RA cohorts. Although the selection of SNPs was based on RA susceptibility studies our results are not related to susceptibility by itself, because no comparison with healthy individuals was done.

Despite the fact that HLA-DRB1*04 alleles are strongly associated with RA and anti-citrulline immunity, few studies have so far addressed the contribution of these allelic variants to RA development via antigen presentation of citrullinated peptides [35–38]. It is not clear why and how HLA-DRB1*04-positive RA patients develop ACPAs with different specificities towards non-overlapping epitopes. It is therefore possible that the HLA-DR*04 alleles (mainly *04:01) together with additional genes outside this region work collectively to shape ACPA responses in RA. In this study, we found three allelic variants outside *HLA-DRB1* that associate with a particular type of autoantibody in RA. These genetic differences may provide a plausible explanation for the phenotypic heterogeneity of RA, for example, differences in the autoantibody profile of RA patients. However, the precise functional contribution of these variants to RA development remains to be determined.

The *PTPN22* gene (MIM ID*600716) encodes an 807 amino acid lymphoid tyrosine phosphatase of non-receptor type (LYP). An SNP in this gene was found to be associated with several autoimmune diseases [39]. This SNP is a non-synonymous change of arginine for tryptophan at position 620 of the protein. The biochemical effect and the resulting disease risk of this SNP have been very difficult to study and there is not yet consensus about its functional consequences. It has been suggested that this SNP determines a gain of function of the LYP phosphatase, but there other studies showing a loss of function of LYP when analyzed together with the c-Src tyrosine kinase [40]. Therefore, it is still unclear how the R620W change might contribute to autoimmunity. In addition, most studies of PTPN22 function refer to T cell function either at the level of thymic selection or of mature T cell activation, but B cells also express LYP and the removal of autoreactive B cells is deficient in subjects bearing the risk allele of *PTPN22*, making for alternative mechanisms of disease risk [41].

The *CDK6* gene (MIM ID* 603368) encodes a 326 amino acid serine/threonine-protein kinase, which is ubiquitously expressed and involved in cell cycle control and differentiation by promoting G1/S transition. A genetic polymorphism at *CDK6* was recently found to be associated with RA [42] and with joint destruction during RA [43]. Interestingly, polymorphisms in this gene are also associated with adult height [44] and with white blood cells counts in some populations [45,46].

The *PADI4* gene (MIM ID *605347) codes for peptidyl arginine deiminase, type 4, which mediates citrullination of different proteins, including histones. In histones, citrullination affects methylation and this way PADI4 influences gene expression [47]. *PADI4* variants have previously been observed to be associated with RA in the Japanese population [48] and recently in European Caucasians [49]. Although the enzyme is directly involved in citrullination of proteins, until now genetic association has not been restricted to RA patients positive for autoantibodies to citrullinated antigens [50].

There were three loci showing significant association with particular autoantibodies in the analysis non-stratified by HLA-DRB1*04. The associations between the anti-citC1^III^ antibodies and the *HLA-DQB1* SNP and between the anti-citVim antibodies and the SNP in *MICA* could reflect linkage disequilibrium with the shared epitope alleles of *HLA-DRB1* because these SNPs are in the HLA region. However, we cannot exclude an effect of other variants in this region as has been shown for RA susceptibility [13]. The third association, between anti-CEP-1 antibodies and the *PTPN22* SNP and anti-CEP-1 antibodies, although observed in the non-stratified analysis was not independent of the presence of HLA-DRB1*04. It was much stronger in the carriers of HLA-DRB1*04 than in the non-carriers, as already discussed.

What are the possible mechanisms underlying the identified associations and how will our findings affect the understanding of, or the management of RA? It is clear that HLA-DRB1*04, as well as other SE alleles, do not represent a single determinant for development of autoimmune responses. We previously demonstrated the possibility of gene-gene and gene-environment interactions in the development of RA which suggested that smoking and several non-HLA genes could serve as possible second determinants for a break in tolerance to self antigens and the development of RA. Two of the genes in our study, *CDK6* and *PTPN22*, are directly involved in signaling pathways that are essential for immune cell function. However, due to redundancy of these pathways we cannot point to the specific type of cells that are involved in the development of disease: it could be downstream mechanisms with T helper cells as primer players, or upstream mechanisms related to antigen presenting cells, B cells, macrophages/monocytes or dendritic cells. As the combination of HLA-DRB1*04 and variations from these genes are unlikely to be sufficient for RA development, one would expect a third determinant to be involved in this interaction. Our findings warrant future studies of the development of autoimmune responses in individuals at risk of RA, with certain subphenotypes, based on serological testing. It would guide both understanding of specific mechanisms and probable treatment towards precision medicine in contrast to current treatment based on relatively non-specific immunosuppressive therapy (that is, RA symptoms). Such studies may guide both understanding of specific mechanisms and future development of more specific therapies for patients from these different subsets. One such option that may be discussed based on our current observations in ACPA-negative RA patients is to test the selective cyclin-dependent kinase 4/6 (CDK4/6) inhibitor (Momilactone B) which is currently being investigated for treatment of colon cancer [51].

There are several strengths in our study. First, we performed analysis in three independent cohorts of RA patients. Second, we used cohorts from two different countries, with different frequencies of autoantibodies, and different types of RA. This variability between sample collections is important for generalization of our findings. Third, our final conclusions were based on the Bonferroni correction and this is a very conservative approach when analyzing correlated variables, such as the four autoantibodies in our study.

There are also some weaknesses in the current study: 1) as the phenotypes we have studied involved a combination of traits, they comprised relatively small numbers of observations even with several thousands of patients included in the study; 2) inclusion of incident cases (EIRA) and established RA (cohorts 2 and 3) may have decreased the power of our study to discover associations that are specific for a particular disease phase; 3) we did not address the contribution of other SE alleles beyond DRB1*04 in our study due to a relatively low frequency of HLA-DRB1*01 and *10 in our cohorts; 4) it also would be beneficial to address the influence of smoking, but we did not have enough statistical power for inclusion of an additional covariate in our analyses.

## Conclusions

Autoimmune diseases display strong association with HLA alleles, but a substantial number of non-HLA variants are also associated at genome-wide level. Specific subphenotype analyses of these associated loci have been rare, and have usually not been stratified by HLA alleles. Our study provides a prototype that could be useful for other studies aiming to disentangle RA complexity. The ongoing replications and extensions of such studies should provide a basis for a molecular re-classification of RA based on genetic and serological features, rather than mainly on clinical data [52]. Specifically, our study confirms the hypothesis that polymorphisms outside the *HLA-DRB1* locus contribute to the development of specific serological subphenotypes of RA, which may have different mechanisms of disease development.

## Additional files

Additional file 1: Table S1. Association between anti-citrullinated cyclic peptide (anti-CCP) and single nucleotide polymorphisms (SNPs) in cohort 1. **Table S2.** Association between anti-Cit-Vimentin and SNP in cohort 1. **Table S3.** Association between anti-citrullinated alpha-enolase peptide-1 (CEP-1) and SNP in cohort 1. **Table S4.** Association between anti-CitC1^III^ and SNP in cohort 1. **Table S5.** Association between anti-CCP and SNP in cohort 2. **Table S6.** Association between anti-Cit-Vimentin and SNP in cohort 2. **Table S7.** Association between anti-CEP1 and SNP in cohort 2. **Table S8.** Association between anti-CitC1 and SNP in cohort 2. **Table S9.** Association between anti-CCP and SNP in cohort 3. **Table S10.** Association between anti-Cit-Vimentin and SNP in cohort 3. **Table S11.** Association between anti-CEP-1 and SNP in cohort 3. **Table S12.** Complete meta-analysis results. **Table S13.** Baseline characteristics of three analyzed RA cohorts. **Table S14.** Genetic variants used in the study. Additional file 2: Figure S1. Levels of autoantibodies in relation to *CDK6*, *PADI4* and *PTPN22* genotypes. Subjects from cohort 1 (upper panel) and cohort 2 (lower panel) were classified negative or positive to *HLA-DRB1*04* and according to their *CDK6*, *PADI4* or *PTPN22* genotypes. Antibody levels against CCP (*CDK6*, *PADI4*) and cit-Vim (*PTPN22*) were compared between the different genotype groups. Bars indicate the median levels of antibody response. ***P <0.05.

## Abbreviations

ACPA: anti-citrullinated peptide antibody
anti-CCP: anti-citrullinated cyclic peptide
CEP-1: citrullinated alpha-enolase peptide-1
citC1^III^: citrullinated type-II collagen trimer
cit-Vim: citrullinated vimentin
CMH test: Cochran-Mantel-Haenszel test
DAS28: disease activity score based on 28 joints
EIRA: epidemiological investigation of rheumatoid arthritis
ELISA: enzyme-linked immunosorbent assay
GWAS: genome-wide association study
HLA: human leucocyte antigen (locus)
LYP: lymphocyte phosphatase
OR: odds ratio
PCR: polymerase chain reaction
RA: rheumatoid arthritis
SE: shared epitope
SNP: single nucleotide polymorphism. Gene names are not listed and follow current genetic nomenclature
