# Supplementary figures and images for: Non-HLA genes PTPN22, CDK6 and PADI4 are associated with specific autoantibodies in HLA-defined subgroups of rheumatoid arthritis
Source: Arthritis Res Ther. 2014 Aug 20;16(4):414. doi: 10.1186/s13075-014-0414-3 (PMC4292996; doi:10.1186/s13075-014-0414-3)

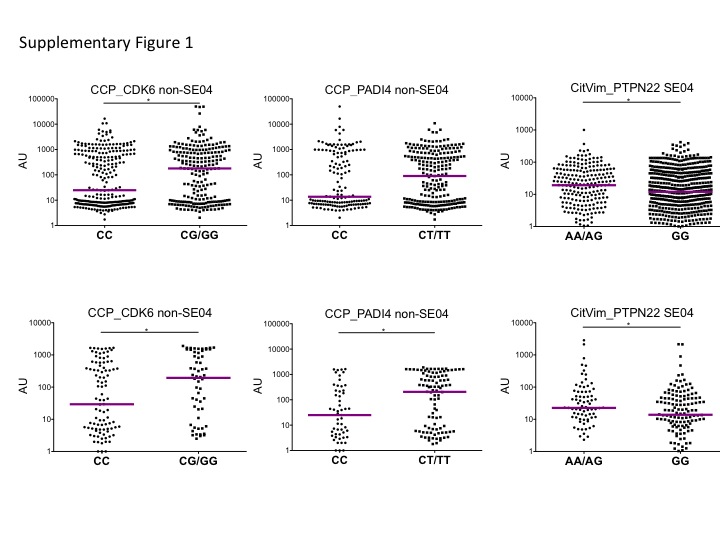

Supplement: Additional file 2: Figure S1. — Levels of autoantibodies in relation to CDK6, PADI4 and PTPN22 genotypes. Subjects from cohort 1 (upper panel) and cohort 2 (lower panel) were classified negative or positive to HLA-DRB1*04 and according to their CDK6, PADI4 or PTPN22 genotypes. Antibody levels against CCP (CDK6, PADI4) and cit-Vim (PTPN22) were compared between the different genotype groups. Bars indicate the median levels of antibody response. *P <0.05. [file 13075_2014_414_MOESM2_ESM.jpeg]
